# The Combination of Schisandrol B and Wedelolactone Synergistically Reverses Hepatic Fibrosis Via Modulating Multiple Signaling Pathways in Mice

**DOI:** 10.3389/fphar.2021.655531

**Published:** 2021-06-03

**Authors:** Yongqiang Ai, Wei Shi, Xiaobin Zuo, Xiaoming Sun, Yuanyuan Chen, Zhilei Wang, Ruisheng Li, Xueai Song, Wenzhang Dai, Wenqing Mu, Kaixin Ding, Zhiyong Li, Qiang Li, Xiaohe Xiao, Xiaoyan Zhan, Zhaofang Bai

**Affiliations:** ^1^ Department of Hepatology, The Fifth Medical Centre, Chinese PLA General Hospital, Beijing, China; ^2^ Research Center for Clinical and Translational Medicine, The Fifth Medical Center of Chinese PLA General Hospital, Beijing, China; ^3^ China Military Institute of Chinese Materia, The Fifth Medical Centre, Chinese PLA General Hospital, Beijing, China

**Keywords:** schisandrol B, wedelolactone, hepatic fibrosis, combined pharmacotherapy, TGF-β1/smads signaling pathway

## Abstract

Hepatic fibrosis represents an important event in the progression of chronic liver injury to cirrhosis, and is characterized by excessive extracellular matrix proteins aggregation. Early fibrosis can be reversed by inhibiting hepatocyte injury, inflammation, or hepatic stellate cells activation, so the development of antifibrotic drugs is important to reduce the incidence of hepatic cirrhosis or even hepatic carcinoma. Here we demonstrate that Schisandrol B (SolB), one of the major active constituents of traditional hepato-protective Chinese medicine, Schisandra sphenanthera, significantly protects against hepatocyte injury, while Wedelolactone (WeD) suppresses the TGF-β1/Smads signaling pathway in hepatic stellate cells (HSCs) and inflammation, the combination of the two reverses hepatic fibrosis in mice and the inhibitory effect of the combination on hepatic fibrosis is superior to that of SolB or WeD treatment alone. Combined pharmacotherapy represents a promising strategy for the prevention and treatment of liver fibrosis.

## Introduction

Hepatic fibrosis is caused by chronic injury and persistent excessive inflammation, which is accompanied with chronic HBV or HCV infection, alcoholic steatohepatitis, NASH, and biliary diseases (Cordero-Espinoza and Huch, 2018). Hepatic fibrosis is a key process in the development of chronic injury to cirrhosis, eventually leading to liver failure and even hepatocellular carcinoma with a worldwide mortality. Once chronic injury or persistent inflammation is resolved, hepatic fibrosis can be regressed (Bansal and Chamroonkul, 2019). Therefore, treatment of hepatic fibrosis is important for the prevention of cirrhosis and related diseases. At present, many potential therapeutic drugs including chemicals and biological drugs have been developed to prevent and treat hepatic fibrosis (Fagone et al., 2016; Schuppan et al., 2018), but there is still a lack of effective antifibrotic drugs in clinic.

Many evidences demonstrated that hepatic stellate cells (HSCs) and macrophages play the key role in driving hepatic fibrogenesis (Bataller and Brenner, 2005; Seki and Schwabe, 2015). Quiescent HSCs can rapidly differentiate into myofibroblast with increased extracellular matrix (ECM) synthesis and deposition in response to fibrogenic stimuli, including transforming growth factor-β1 (TGF-β1) and platelet-derived growth factor (PDGF) (Liu et al., 2006; Inagaki and Okazaki, 2007), so activated HSCs are central to the pathogenesis of hepatic fibrosis. Chronic injury-mediated macrophages recruitment and activation may promote the survival of activated HSCs *via* Toll-like receptor 4 (TLR4)-dependent production of chemokines and pro-inflammatory cytokines, such as interleukin-1β (IL-1β) and tumor necrosis factor-α (TNF-α) (Pradere et al., 2013). If the recruited inflammatory cells are not effectively resolved, they can further exacerbate the tissue injury, thereby resulting in hepatic fibrosis (Krenkel et al., 2018). As TGF-β1 has been considered as the key cytokine in the activation of HSCs, multiple small molecules or antibodies that target TGF-β1 have potential value in prevention and treatment of hepatic fibrosis (Fagone et al., 2016). But TGF-β1 is a multifunctional cytokine with broad biological activities involving multiple biological functions, such as embryogenesis, immunity, carcinogenesis and inflammation (Wynn and Ramalingam, 2012; Wu et al., 2017), whether long-term inhibition of TGF-β1 activity is beneficial to patients with hepatic fibrosis remains to be further studied. Some studies have demonstrated that the combination of small molecules has been successfully used in the treatment of liver fibrosis in animal models (Yang et al., 2012; Sung et al., 2018), suggesting that combined pharmacotherapy will represent a promising strategy for the prevention and treatment of hepatic fibrosis.

Some Traditional Chinese Medicine (TCM) formulations have been approved by the China Food and Drug Administration and are widely used for the treatment of hepatic fibrosis, such as Biejiaruangan Compound and Fuzheng Huayu (FZHY) (Zhang and Schuppan, 2014). Besides, the herbal prescription Yang-Gan-Wan and its active compound polyphenolic rosmarinic acid combined with baicalin has also been proved to be able to treat hepatic fibrosis (Yang et al., 2012). Liuweiwuling tablets (LWWL) is a Chinese Medicine formula which is composed of six herbs: *Schisandrae chinensis fructus, Fructus Ligustri Lucidi, Forsythiae fructus, Curcumae rhizoma, Perennial sow thistle, and Ganoderma spore* and is well known for protection against liver injury in patients with chronic hepatitis B in China (Xin et al., 2009; Lei et al., 2015; Du and Jaeschke, 2016). Clinical studies have also confirmed that LWWL also has definite therapeutic effects on liver fibrosis, we also demonstrated that LWWL could attenuate hepatic fibrosis via modulation of TGF-β1 and NF-κB signaling pathways in BDL and CCL4-induced hepatic fibrosis rat models (Liu et al., 2018a; Liu et al., 2018b). Schisandrol B (SolB) is one of the main active ingredients isolated from *Schisandrae chinensis fructus*, and has been shown to have definite hepatoprotective effects in APAP and cholestasis induced liver injury models (Jiang et al., 2015; Zeng et al., 2017). Wedelolactone (WeD) is a coumarin isolated from Eclipta prostrate L, which exhibits anti-inflammatory effects (Yuan et al., 2013; Luo et al., 2018; Zhu et al., 2019), it also has anti-fibrotic effects on human hepatic stellate cell line LX-2 (Xia et al., 2013). In our study, we demonstrate that the combination of SolB and WeD synergistically reverses hepatic fibrosis *via* modulating multiple signaling pathways, suggesting that the combination of SolB and WeD may be developed as potential candidate for the treatment of liver fibrosis.

## Materials and Methods

### Mice

Six-to-eight-week-old female C57BL/6 mice were purchased from SPF Biotechnology Co., Ltd. (Beijing, China). All animals were maintained under 12 h light/dark conditions at 22–24°C with unrestricted access to food and water for the duration of the experiment. All animal protocols in this study were performed according to the guidelines for care and use of laboratory animals and approved by the animal ethics committee of the Fifth Medical Center, Chinese PLA General Hospital (Beijing, China).

### Reagents and Antibodies

Nigericin, dimethyl sulfoxide, and ultrapure lipopolysaccharide (LPS) were purchased from Sigma (Munich, Germany). SolB, WeD, and colchicine were obtained from TargetMol (Boston, MA, United States). Anti-mouse-Collagen1 (1:1000), anti-mouse-Smad2/3 (1:1000), and anti-mouse-P-Smad3 (1:1000) were purchased from Cell Signaling Technology (Boston, MA, United States). Anti-mouse-P-IκBα (1:1000), anti-mouse-IκBα (1:1000), anti-mouse -P-IKK (1:1000), anti-mouse-IKK (1:1000), anti-human Smad3 (1:1000), anti-human P-Smad3 (1:1000), and anti-human *α*-SMA (1:1000) were purchased from Proteintech (Chicago, IL, United States). Anti-mouse-IL-1β, anti-mouse-NLRP3 (1:2000), and anti-mouse-ASC (1:1000) were purchased from Santa Cruz Biotechnology (Beijing, China). Anti-mouse -caspase-1 p20 (1:1000), anti-mouse-caspase-1 p45 (1:1000), anti-mouse-pro-IL-1β (1:1000), and anti-GAPDH (1:2000) were purchased from Proteintech (Chicago, IL, United States).

### Cell Culture

Bone marrow-derived macrophages (BMDMs) were isolated from the femoral bone marrow of 10°week-old female C57BL/6 mice and cultured in Dulbecco’s modified Eagle’s medium (DMEM) supplemented with 10% fetal bovine serum (FBS), 1% penicillin/streptomycin (P/S), and 50 ng/ml murine macrophage colony-stimulating factor. LX-2 and L0-2 cells were grown in RPMI 1640 medium. All cell lines were cultured under a humidified 5% (v/v) CO_2_ atmosphere at 37°C.

### Animal Experiments

Four-to-five-week-old female C57BL/6 mice were purchased from SPF Biotechnology Co., Ltd. (Beijing, China). All animals were maintained under 12 h light/dark conditions at 22–24°C with unrestricted access to food and water for the duration of the experiment, except during fasting tests. All animal protocols in this study were performed according to the guidelines for care and use of laboratory animals and approved by the animal ethics committee of the Fifth Medical Center, Chinese PLA General Hospital. For experiments with BDL, mice were randomly divided into six groups. From days 7 to 21 after surgery, the sham group and the mice with successful development of the disease were divided into groups and administered drugs by gavage with a dose of 2 ml/kg once a day. The mice were divided into the following groups: sham operation group (Control), model group (BDL/CCl_4_-induced hepatic fibrosis model group), colchicine positive group (0.2 mg/kg), WeD 20 mg/kg group, SolB 40 mg/kg group, and WeD 20 mg/kg in combination with SolB 40 mg/kg group. Eight mice were included in each group. The body weight was measured daily. The animals were euthanized in random order between 9:00 am and 11:00 am after an overnight fast. Samples of serum and liver were collected for further analysis.

### Serum Biochemistry and Liver Histology

Alanine transaminase (ALT) and aspartate transaminase (AST) were analyzed using kits from Thermo Fisher Scientific (Cincinnati, OH, United States). Formalin-fixed tissue was embedded in paraffin, and sections were stained with Masson, hematoxylin and eosin (H&E), and Sirius red stains. Liver histology was blindly assessed for inflammation, necrosis, and bile duct proliferation. Hydroxyproline levels in the liver were measured as described.

### Quantitative Real-Time Polymerase Chain Reaction

qPCR method was used to detect gene expression in liver tissue of mice in each group. After RNA extraction, Reverse transcription kit was used to reverse transcription RNA cDNA, and the specific operation was carried out strictly in accordance with the kit instructions. Using a qPCR instrument to amplify each gene and its GAPDH, qPCR reaction system: cDNA1μL, SYBR Green Mix 5 , 0.5 μl of upstream and downstream primers, DEPC water 3 μl. Reaction conditions 95°C 3 min, 95°C 3 s, 60°C 30 s, 95°C 15 s, 60°C 1 min, 95°C 15 s, Cycle 50 times. Each sample contains two multiple holes, gene expression levels were quantitatively analyzed by 2-ΔΔCt method.

### Western Blotting

Immunoblot analysis was used to evaluate the expression of Collagen1, *p*-Smad3, Smad2/3, *p*-IκB, IκBα, IKK, *p*-IKK, NLRP3, Lamin B, *α*-SMA, caspase-1 p20, IL-1β p17, pro-IL-1β, caspase-1 p45, NLRP3, and ASC. GAPDH and Lamin B served as loading controls in cell lysates. The samples were boiled at 105°C for 15 min. The protein samples were resolved using 12 or 10% sodium dodecyl sulfate-polyacrylamide gel electrophoresis (SDS-PAGE) gels and transferred to a nitrocellulose membrane *via* a wet-transfer system. The membranes were subsequently incubated with 5% fat-free milk for 1 h at room temperature followed by incubation overnight with primary antibodies at 4°C. Blots were washed thrice with Tris-buffered saline Tween-20 (TBST) and incubated with corresponding horseradish peroxidase-conjugated secondary antibody (1:5000) for 1 h at room temperature. This step was followed by washing with TBST thrice, and the signals were analyzed using the enhanced chemiluminescent reagent (Promega, Beijing, China) detection system.

### Infammasome Activation

BMDMs were seeded at 5×10^5^ cells/well in 24-well plates overnight. The following day, the medium was replaced, and cells were stimulated with 50 ng/ml LPS for 4 h. The medium was then changed to opti-MEM containing WeD or SolB for 1 h. For inducing the activation of NLRP3 infammasome, cells were stimulated with nigericin (7.5 μmol/L) for 45 min.

### NF-κB Signing Pathway Activation

BMDMs were seeded at 5×10^5^ cells/well in 24-well plates overnight. BMDMs were treated with WeD or SolB for an hour and then stimulated with LPS (50 ng/ml) for 0, 5, 15, 30, and 60 min.

### TGF-β1 in Combination With LPS Induced Hepatocyte Injury *in vitro*

We seeded BMDMs at 5×10^5^ cells/well in 24-well plates overnight. The medium was then replaced with 1640 medium without FBS, then give SolB or WeD for an hour, followed by treatment with TGF-β1 (5 ng/ml) alone or in combination with LPS (50 ng/ml).

### Cell Viability Assay

The cell counting kit-8 (CCK-8) assay was used to detect the viability of cells. L0-2 cells were seeded in a 96-well growth-medium plate overnight at 8.5×10^4^ cells/well. The cells were then incubated at 37°C followed by individual treatments with WeD or SolB for 24 h. These cells were incubated with CCK-8 for 30 min. The optical density (O.D.) values were determined at the wavelength of 450 nm. The half-maximal inhibitory concentration (IC50) of WeD or SolB was evaluated using the software Prism 6 (GraphPad Software, San Diego, CA, United States).

### APAP-Induced Hepatocyte Injury *in vitro*

To induce hepatocyte injury, L0-2 cells were seeded at 8.5×10^3^ cells/well in 96-well plates overnight. The following day, the medium was replaced with DMEM containing APAP (22 mmol/L) and SolB or WeD (5, 10, 20, 40 μmol/L).

### Enzyme-Linked Immunosorbent Assay

Supernatants from cell culture were assayed with mouse TNF-α and mouse IL-6 according to manufacturer's instructions (Dakewe, Beijing, China).

### Lactate Dehydrogenase Assay

L0-2 cells were treated with WeD and SolB separately. The release of LDH into the culture supernatants was evaluated using the LDH cytotoxicity assay kit (Beyotime, Shanghai, China) according to the manufacturer’s instructions.

### Statistical Analysis

Statistical analysis was performed using the software Prism 6 (GraphPad Software, San Diego, CA, United States). All experimental data were expressed as mean ± standard error of the mean (SD). One-way ANOVA was used for multiple comparisons followed by Tukey’s *post hoc* test or Dunnett’s test for comparison between two groups. The differences with *p* < 0.05 were considered statistically significant.

## Results

### SolB Significantly Inhibited Hepatocyte Injury *in vitro*

**FIGURE 1:**
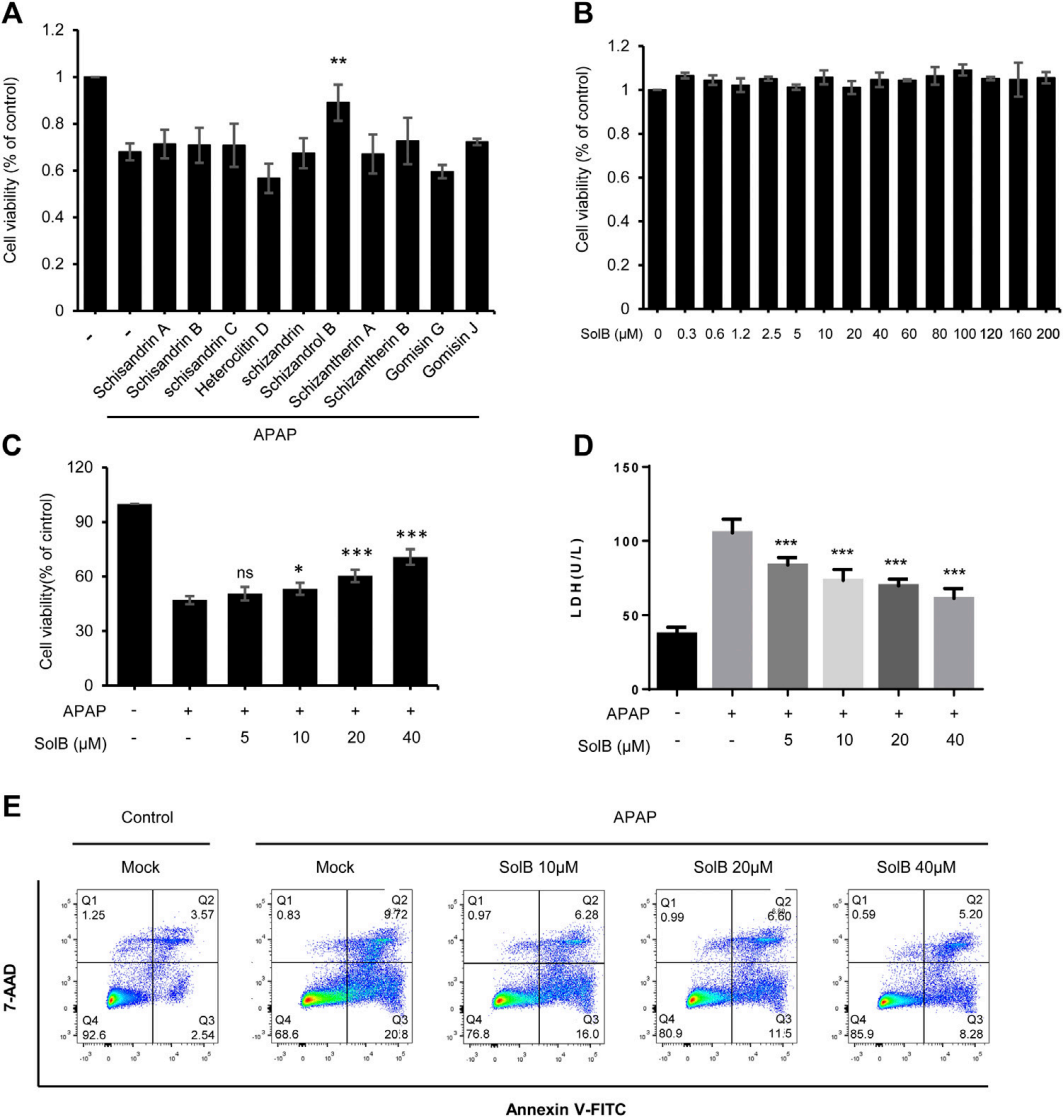
SolB significantly inhibited hepatocyte injury *in vitro* **(A)** The effect of the constituents of *Schisandra sphenanthera* on APAP-induced hepatocyte injury **(B)** Cell viability of L02 cells treated with SolB for 24 h were detected **(C,D)** Survival rate by CCK-8 **(C)** and the release of LDH **(D)** of L02 cells treated with SolB and then exposed to APAP **(E)** Early apoptotic of L02 cells were treated with SolB and then exposed to APAP, and detected by Flow cytometry. Data are expressed as Mean ± SD from three biological replicates (*n* = 3). Statistics differences were analyzed using One-way ANOVA followed by Dunnett’s test:*p < 0.05, **p < 0.01, ***p < 0.001; NS, no significance.

Persistent hepatocyte injury or death, as an main driver of fibrosis and liver inflammation, could initiate and perpetuate sterile inflammatory responses and HSCs activation, so hepatic fibrosis can be reversed by inhibiting hepatocyte injury or eliminating the triggering factors (Cordero-Espinoza and Huch, 2018; Bansal and Chamroonkul, 2019). *S. sphenanthera* is a traditional hepato-protective Chinese medicine, so we test the effect of the constituents of *S. sphenanthera* on APAP-induced hepatocyte injury. The data showed that several constituents could improve cell viability of LO-2 cells exposed to acetaminophen, and Schisandrol B (SolB) is the most effective constituent (Figure 1A). We then tested the effect of the SolB on hepatocyte survival by the CCK8 assay and the result showed that SolB were not toxic to LO-2 cells, even at concentrations up to 200 μmol/L (Figure 1B). We next examined whether SolB inhibited hepatocyte injury at a wide range of concentrations (0, 5,10, 20, and 40 μM). The result showed that SolB treatment dose-dependently improved cell viability and inhibited LDH release in LO-2 cells exposed to acetaminophen (Figures 1C,D). The protective effects of SolB on hepatocytes was further examined using Annexin V-FITC Apoptosis detection kit, the result showed that SolB treatment significantly inhibited acetaminophen-induced apoptosis of LO-2 cells (Figure 1E). These results indicated the protective effect of SolB against hepatocyte injury.

### SolB Attenuates Hepatic Injury and Fibrosis Induced by BDL in Mice

**FIGURE 2:**
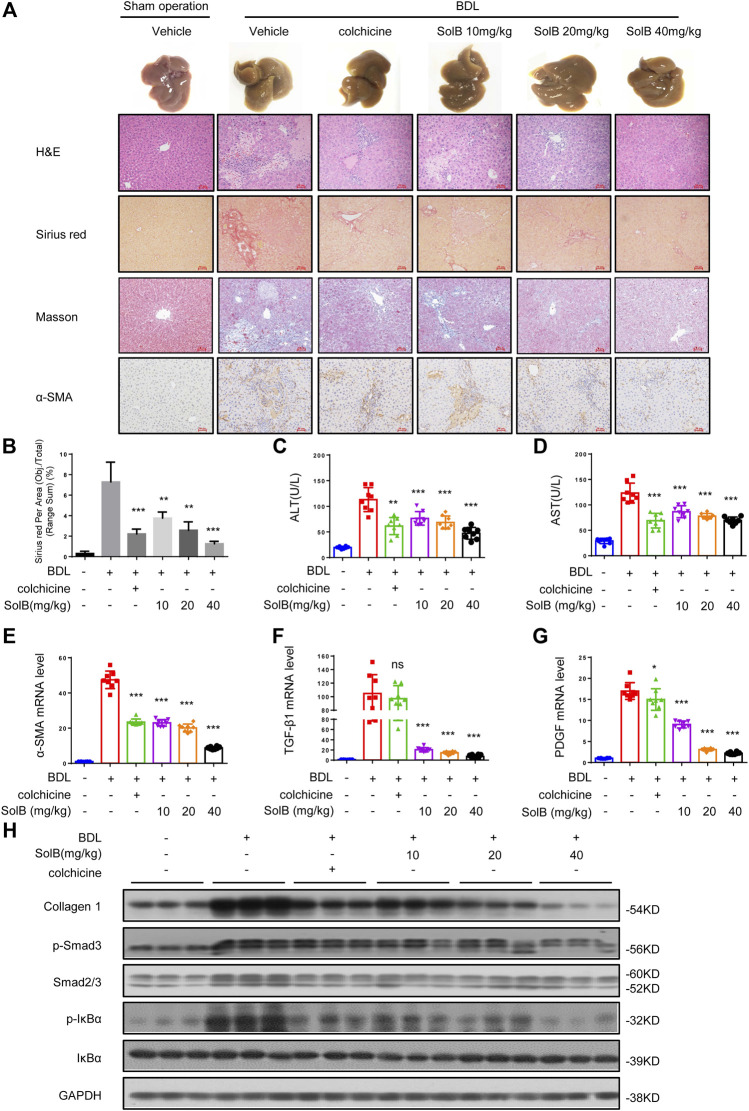
SolB attenuates hepatic injury and fibrosis induced by BDL in mice **(A)** Images of livers from sham-operated mice (SHAM), bile duct-ligated mice (BDL), BDL-mice treated with SolB. Representative micrographs of liver H&E staining, Sirius red, Masson staining and *α*-SMA were shown. Scale bars represent 50 μm **(B)** Quantitative results of Sirius red staining sections **(C,D)** Serum level of ALT **(C)** and AST **(D)** of sham-operated mice (SHAM), bile duct-ligated mice (BDL) and BDL-mice treated with SolB or colchicine (0.2 mg/kg) **(E–G)** Quantitative PCR analysis of mRNA levels of *α*-SMA **(E)**, TGF-β1 **(F)**, and PDGF **(G)** in livers from sham-operated mice, BDL mice, BDL-mice treated with SolB **(H)** Western blot analysis of Collagen1, *p*-Smad3, Smad2/3, P-IκBα, IκBα, and GAPDH in livers from sham-operated mice, BDL mice, BDL-mice treated with SolB. Data are expressed as Mean ± SD (*n* = 8 or 3 mice). Statistics differences were analyzed using One-way ANOVA followed by Dunnett’s test: *p < 0.05, **p < 0.01, ***p < 0.001. NS, no significance.

Considering the obviously hepato-protective effect, we tested the role of SolB in mouse model of hepatic fibrosis induced by bile duct ligation (BDL) ligation. The livers of mice treated with colchicine (colchicine has been used for the treatment of liver fibrosis (Rambaldi and Gluud, 2005) and was used as a positive control in this study, 0.2 mg/kg per day) or SolB (10, 20, and 40 mg/kg per day, respectively) exhibited a remarkably lower cirrhotic appearance with varying degrees (Figures 2A–D). Furthermore, the histological assessment of liver sections by H&E staining, Sirius red, and Masson staining demonstrated that treatment with SolB inhibited hepatic injury and fibrosis in mice induced by BDL (Figures 2A,B). Consistent with the histopathology analysis, SolB treatment reversed the increase in ALT, AST, DBIL, TBIL, and TBA serum levels induced by BDL in a dose-dependent manner in mice (Figures 2C,D; Supplementary Figures S1A–C).

HSCs play a crucial role in hepatic fibrosis, which can be activated upon persistent liver injury and leads to the deposition of ECM components including collagen 1 (Shang et al., 2018). The *α*-smooth muscle actin (α-SMA) is a unique marker of activated HSCs (Nouchi et al., 1991). Hence, we analyzed the expression of *α*-SMA by immunohistochemical staining (Figure 2A) and qPCR (Figure 2E) in all groups. The results demonstrated that SolB inhibited the expression of *α*-SMA in mice with hepatic fibrosis induced by BDL in a dose-dependent manner. Our results also showed that treatment with SolB reduced the number of macrophages, staining by specific marker of F4/80, in mice induced by BDL. Consequently, this observation indicated that SolB inhibited the activation of HSCs *in vivo.* Activation and survival of HSCs are regulated by transforming growth factor-beta (TGF-β) and platelet-derived growth factor (PDGF) signaling pathways (Pinzani 2002; Kikuchi et al., 2017; Dewidar et al., 2019). The expression of TGF-β and PDGF was also inhibited by SolB treatment in mice with hepatic fibrosis induced by BDL, as evidenced by qPCR (Figures 2F,G). BDL significantly promoted collagen I gene expression compared to that in the normal mice, while SolB treatment significantly abrogated BDL-induced upregulation of collagen I, as revealed by immunoblotting (Figure 2H; Supplementary Figure S1D) and qPCR analysis (Supplementary Figure S1E). More importantly, we observed that SolB inhibited both the phosphorylation of Smad3 and the expression of Smad2/3 in a dose-dependent manner (Figure 2H; Supplementary Figure S1E). We also have evaluated macrophages (by F4/80) in the liver of the BDL-induced hepatic fibrosis mice. Our results showed that treatment with SolB reduced the number of macrophages, staining by specific marker of F4/80, in mice induced by BDL (Supplementary Figure S1F). These results demonstrated that SolB attenuates hepatic injury and fibrosis induced by BDL in mice*.*

### WeD Inhibits TGF-β1/Smad-Mediated Activation of HSCs *in vitro*

**FIGURE 3:**
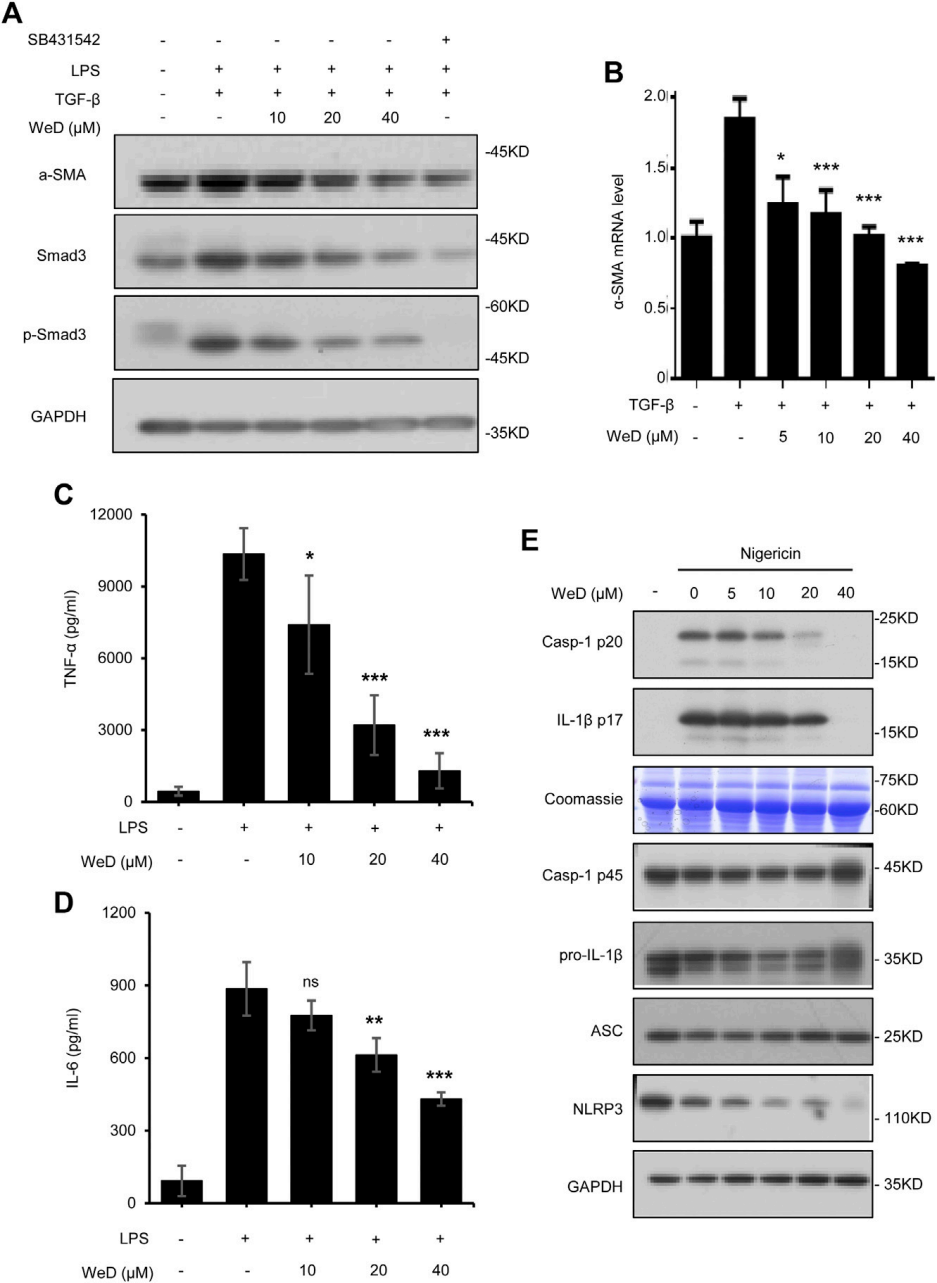
WeD inhibits TGF-β1/Smad-mediated activation of HSCs and blocks the production of TNF-α and IL-1β in macrophages **(A)** Western blot analysis of *α*-SMA, Smad3, *p*-Smad3, GAPDH in LX-2 cells treated with WeD (10, 20, 40 μM) and then stimulated with TGF-β1 (5 ng/ml) combined with LPS (50 ng/ml) **(B)** Quantitative PCR analysis of mRNA levels of *α*-SMA of LX-2 cells treated with WeD and then stimulated with TGF-β1 (5 ng/ml) **(C,D)** ELISA of TNF-α **(C)** and IL-6 **(D)** in SN of BMDMs treated with WeD (10, 20, 40 μM) and then stimulated with LPS (50 ng/ml) **(E)** Western blot analysis of caspase-1 (p20) and IL-1β in SN and pro- IL-1β, caspase-1 (p45), NLRP3 and ASC in WCL of LPS-primed BMDMs treated with WeD (5, 10, 20, 40 μM) and then stimulated with nigericin. Data are expressed as Mean ± SD from three biological replicates (*n* = 3). Statistics differences were analyzed using One-way ANOVA followed by Dunnett’s test: *p < 0.05, **p < 0.01, ***p < 0.001. NS, no significance.

Combination therapy for liver fibrosis is considered to be very attractive. Targeting several vital but very different pathways to reduce chronic inflammation and ECM deposition would more effectively address liver fibrosis (Schuppan and Kim, 2013; Schuppan, 2015). So we reasoned that the combination of SolB with another compound that target HSC activation would exhibit better anti-fibrosis effect. Activated HSCs play a central role in the pathogenesis of hepatic fibrogenesis, in which a continuous synthesis of collagen is mediated by the TGF-β1/Smad signaling pathway (Lee and Friedman, 2011). We screened compounds that could inhibit TGF-β1/Smad signaling in LX-2 cells (human hepatic stellate cell line) and found that Wedelolactone (WeD) could suppress TGF-β1/Smad pathway (Supplementary Figures S2A,B). Next we tested whether WeD could inhibit TGF-β1/Smad signaling to suppress the activation of HSCs *in vitro*. We detected the expression and phosphorylation of Smad3 in the TGF-β1 signaling cascade. The result demonstrated a reduction in the expression of Smad3 and its phosphorylation after treatment with WeD in LX-2 cells pretreated with TGF-β1 (Figure 3A; Supplementary Figure S2C). WeD also inhibited TGF-β1-mediated induction of *α*-SMA (Figures 3A,B; Supplementary Figure S2C). These results demonstrated that WeD could suppress the activation of HSCs by targeting the TGF-β1/Smad signaling pathway.

### WeD Blocks the Production of TNF-α and IL-1β in Macrophages

Previous studies have demonstrated that TNF-α and IL-1β produced by hepatic macrophages can promote the survival of activated HSCs (Gieling et al., 2009; Osawa et al., 2013; Pradere et al., 2013). Wedelolactone has been reported to be an inhibitor of IKK that is critical for activation of NF-κB by mediating phosphorylation and degradation of IκBα (Kobori et al., 2004), activation of NF-κB signaling leads to expression of downstream target genes including TNF-α and IL-6. Hence, we next tested whether WeD affected the production of TNF-α and IL-6 in LPS-treated macrophages, the result showed that WeD dose-dependently inhibited the production of TNF-α and IL-6 in LPS-treated BMDMs (Figures 3C,D). The maturation and release of IL-1β are mediated by the inflammasome. WeD has been reported to inhibit the activation of the NLRP3 inflammasome (Wei et al., 2017; Cheng et al., 2019). We further confirmed the effect of WeD on NLRP3 inflammasme activation and release of IL-1β. Our data showed that WeD inhibited nigericin-induced caspase-1 cleavage and IL-1β production in a dose-dependent manner in LPS-primed BMDMs (Figure 3E; Supplementary Figure S2D), suggesting that WeD inhibited the production of IL-1β by directly blocking the activation of the NLRP3 inflammasome. Taken together, these results demonstrated that WeD can directly inhibit the production of TNF-α and IL-1β in macrophages.

### WeD Attenuates Hepatic Injury and Fibrosis Induced by BDL in Mice

**FIGURE 4:**
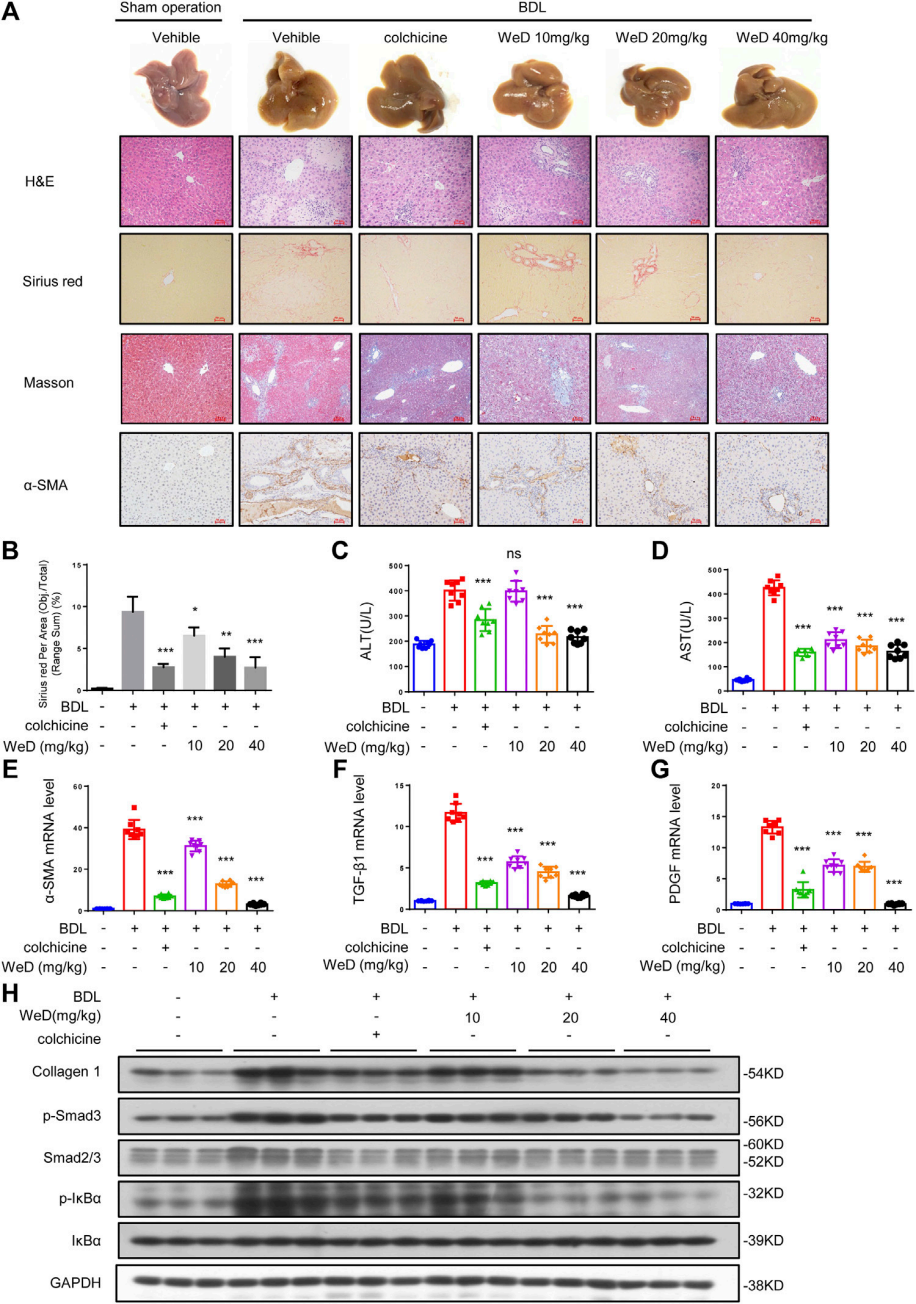
WeD attenuates hepatic injury and fibrosis induced by BDL in mice **(A)** Images of livers from sham-operated mice (SHAM), bile duct-ligated mice (BDL), BDL-mice treated with WeD. Representative micrographs of liver H&E staining, Sirius red, Masson staining, and *α*-SMA were shown. Scale bars represent 50 μm **(B)** Quantitative results of Sirius red staining sections **(C,D)** Serum level of ALT **(C)** and AST **(D)** of sham-operated mice (SHAM), bile duct-ligated mice (BDL) and BDL-mice treated with WeD or colchicine (0.2 mg/kg) **(E–G)** Quantitative PCR analysis of mRNA levels of *α*-SMA **(E)**, TGF-β1 **(F)**, and PDGF **(G)** in livers from sham-operated mice, BDL mice, BDL-mice treated with WeD **(H)** Western blot analysis of Collagen1, *p*-Smad3, Smad2/3, P-IκBα, IκBα and GAPDH in livers from sham-operated mice, BDL mice, BDL-mice treated with WeD. Data are expressed as Mean ± SD (*n* = 8 or 3 mice). Statistics differences were analyzed using One-way ANOVA followed by Dunnett’s test: *p < 0.05, **p < 0.01, ***p < 0.001. NS, no significance.

Considering the inhibitory effect of WeD on HSC activation and inflammation, we further tested the role of WeD in liver fibrosis in a mouse model of hepatic fibrosis induced by BDL. Our result of histological assessment of liver sections by H&E staining, Sirius red, and Masson staining showed that treated with WeD significantly alleviated hepatic injury and fibrosis in BDL-induced mice (Figures 4A,B). Consistent with the histopathology analysis, WeD treatment reduced the ALT, AST, DBIL, TBIL, and TBA serum levels induced by BDL in a dose-dependent manner in mice (Figures 4C,D; Supplementary Figure S3A–C).

It has been reported that HSCs play a crucial role in hepatic fibrosis and *α*-SMA is a unique marker of activated HSCs (Nouchi et al., 1991; Shang et al., 2018). So we detected the expression of *α*-SMA by immunohistochemical staining and qPCR. Our result showed that the expression of *α*-SMA in mice with hepatic fibrosis induced by BDL could be reduced by WeD treatment (Figures 4A,E). Our results showed that treatment with WeD reduced the number of macrophages, staining by specific marker of F4/80, in mice induced by BDL. Consequently, this observation demonstrated that WeD also inhibited the activation of HSCs *in vivo.* Then we examined the expression of TGF-β and PDGF in mice. The data showed that the expression of TGF-β and PDGF was also inhibited by WeD treatment with BDL-induced hepatic fibrosis by qPCR (Figures 4F,G). WeD treatment also significantly reduced BDL-induced increase of collagen I, as revealed by immunoblotting (Figure 4H; Supplementary Figure S3H) and qPCR analysis (Supplementary Figure S3D). Consistent with the effect *in vitro*, WeD inhibited both the phosphorylation of Smad3 and the expression of Smad3 in a dose-dependent manner (Figure 4H; Supplementary Figure S3H). we have evaluated macrophages (by F4/80) in the liver of the BDL-induced hepatic fibrosis mice. Our results showed that treatment with WeD reduced the number of macrophages, staining by specific marker of F4/80, in mice induced by BDL (Supplementary Figure S3G). We have demonstrated that WeD dose-dependently inhibited the production of TNF-α and IL-6 in LPS-treated BMDMs and the production of IL-1β by directly blocking the activation of the NLRP3 inflammasome in BMDMs. Consistent with the effect in BMDMs, WeD treatment could inhibit the expression of IL-β and IL-6 (Supplementary Figure S3E,F). The results demonstrated that WeD could attenuate inflammation in liver *in vivo*. Thus, these results demonstrated that WeD attenuates hepatic injury and fibrosis induced by BDL in mice and inhibited the TGF-β/Smad-mediated activation of HSCs *in vivo.*

### A Combination of SolB and WeD Significantly Inhibits Hepatic Fibrosis and Injury in Mice With CCl_4_-Induced Hepatic Fibrosis

**FIGURE 5:**
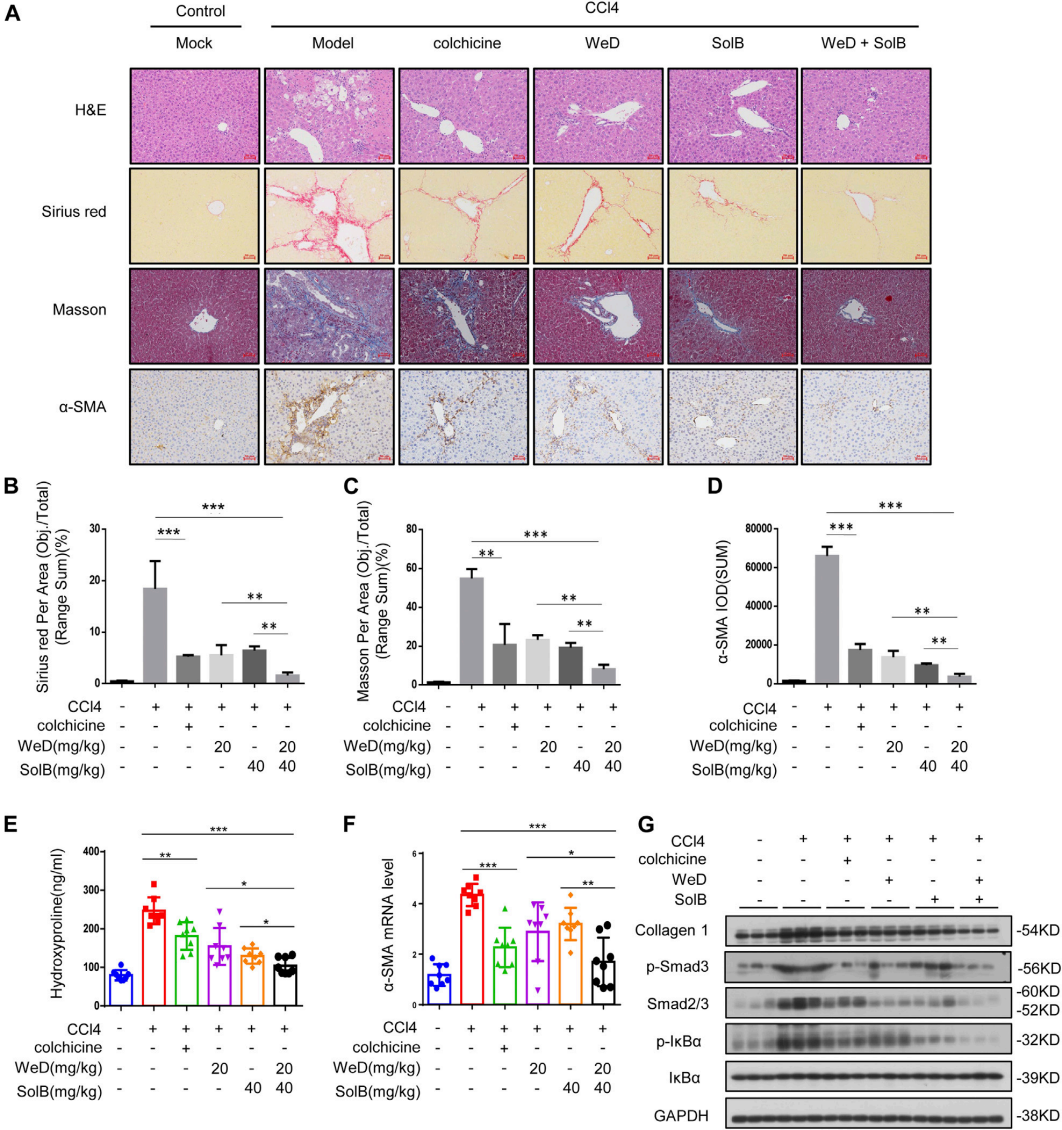
A combination of SolB and WeD treatment dramatically inhibits hepatic fibrosis and injury in CCL4-induced hepatic fibrosis mice **(A)** Images of livers from control, CCL4-induced hepatic fibrosis mice, CCL4-induced hepatic fibrosis mice treated with colchicine (0.2 mg/kg), SolB (40 mg/kg), WeD (20 mg/kg) or combination of SolB (40 mg/kg) and WeD (20 mg/kg). Representative micrographs of liver H&E staining, Sirius red, Masson, and *α*-SMA staining were shown. Scale bars represent 50 μm **(B–D)** Quantitative results of Sirius red **(B)**, Masson **(C)**, and *α*-SMA **(D)** staining sections **(E)** Serum level of hydroxyproline of control, CCL4-induced hepatic fibrosis mice, CCL4-induced hepatic fibrosis mice treated with colchicine (0.2 mg/kg), SolB (40 mg/kg), WeD (20 mg/kg) or combination of SolB and WeD **(F)** Quantitative PCR analysis of mRNA levels of *α*-SMA in livers from control, CCL4-induced hepatic fibrosis mice, CCL4-induced hepatic fibrosis mice treated with colchicine (0.2 mg/kg), SolB (40 mg/kg), WeD (20 mg/kg) or combination of SolB and WeD. **(G)** Western blot analysis of Collagen1, *p*-Smad3, Smad2/3, *p*-IκBα, IκBα and GAPDH in livers from control, CCL4-induced hepatic fibrosis mice, CCL4-induced hepatic fibrosis mice treated with colchicine (0.2 mg/kg), SolB (40 mg/kg), WeD (20 mg/kg) or combination of SolB and WeD. Data are expressed as Mean ± SD (*n* = 8 or 3 mice). Statistics differences were analyzed using One-way ANOVA followed by Tukey’s *post hoc* test: *p < 0.05, **p < 0.01, ***p < 0.001. NS, no significance.

Next we examined if SolB and WeD would show protective effects in another hepatic fibrosis mouse model. We evaluated the anti-fibrosis effect of SolB and WeD in CCl4-induced hepatic fibrosis model mice, and the data showed that both SolB and WeD alleviated hepatic fibrosis induced by CCl_4_ in mice, suggesting their potent anti-fibrosis effect. Next we tested whether the anti-fibrosis effect mediated by the combination of SolB and WeD was more potent in comparison with that mediated by individual treatments of SolB and WeD. Mice with CCl_4_-induced hepatic fibrosis were randomly divided into five groups and received gavages with PBS (model group), colchicine (a positive control drug), 20 mg/kg WeD, 40 mg/kg SolB, or a combination of WeD (20 mg/kg) and SolB (40 mg/kg). Compared with control mice, mice with CCl_4_-induced hepatic fibrosis showed serve hepatocyte damage and hepatic fibrosis, evidenced by HE, Sirius red staining, and Masson staining (Figures 5A–C). WeD, SolB and their combination treatment were all shown to prevent the progression of liver fibrosis in the mouse model with CCl_4_-induced hepatic fibrosis. The synergistic inhibitory effect of SolB and WeD on hepatic fibrosis was significantly better than that of WeD or SolB (Figures 5A–C). Consistent with the results of liver histology assessment, combined treatment of WeD and SolB also more efficiently reduced the hydroxyproline content in liver tissues than WeD or SolB alone (Figure 5E).

To further examine the antifibrotic effect of SolB combined with WeD, the expression of *α*-SMA was evaluated by immunohistochemistry, results showed that CCl_4_ significantly promoted *α*-SMA expression in the portal area of fibrotic liver tissue, while treatment with WeD, SolB, or their combination suppressed the expression of *α*-SMA in fibrotic liver tissues (Figures 5A,D). Furthermore, the expression of *α*-SMA in mice co-treated with SolB and WeD was significantly lower than that in mice treated with WeD or SolB alone (Figures 5A,D). Moreover, compared with the individual WeD or SolB treatment, the combined treatment of WeD and SolB more effectively reduced the mRNA level of *α*-SMA in liver tissues of mice (Figure 5F). Previous studies have shown that cytoglobin, which is the fourth globin in mammals and function as a local gas sensor, is a promising new marker that discriminates between myofibroblasts derived from stellate cells and those from portal fibroblasts (Kawada, 2015; Thuy et al., 2015). Thus, we evaluated the cytoglobin by qPCR in CCl4-induced hepatic fibrosis mice, and the results showed that combination treatment of WeD and SolB significantly reduced the expression of cytoglobin induced by CCl4 in mice compared to that by treatment with WeD and SolB alone, suggesting that the combination of WeD and SolB is more effective in the treatment of liver fibrosis (Supplementary Figure S4A). Western blot analysis also demonstrated that combination treatment of WeD and SolB significantly decreased the expression of collagen and Smad as well as phosphorylation of IκB and Smad3 induced by CCl_4_ in mice compared to that by treatment with WeD and SolB alone, suggesting that WeD combined with SolB could inhibit the activity of the TGF-β1 and NF-κB pathway more effectively than WeD or SolB alone *in vivo* (Figure 5G; Supplementary Figure S4B). These results indicate that the combination of WeD and SolB is more effective in the treatment of liver fibrosis owing to the regulation of several signaling pathways associated with hepatic fibrosis.

## Discussion

In this study, we demonstrate that SolB, WeD or the combination of SolB and WeD could reverse hepatic fibrosis *in vivo* and the inhibitory effect of combination of SolB and WeD on hepatic fibrosis is superior to that of SolB or WeD treatment alone. SolB reverses hepatic fibrosis by inhibiting hepatocyte injury, and WeD could block the production of TNF-α and IL-1β in macrophages and the activation of TGF-β1/Smad signaling pathway in activated HSCs. Our result confirms that combination pharmacotherapy targeting several vital but very different pathways to reduce chronic inflammation and ECM deposition would more effectively address liver fibrosis.

Schisandra sphenanthera is the dried ripe fruit of S. sphenanthera Rehd. et Wils, it is a traditional Chinese medicine widely used for its protective effects in liver, kidney, and heart (Panossian and Wikman, 2008). Recent studies report that Schisandra sphenanthera possesses hepatoprotective effects against viral or chemical hepatitis (Zhu et al., 2000; Teraoka et al., 2012). Hepatocyte injury, a primary inducer of hepatic fibrosis, can promote inflammation and activation of HSCs in liver, therefore, reversal of hepatocyte injury is one of the important ways to prevent and treat hepatic fibrosis (Lee et al., 2015). Our result showed that SolB is the most effective constituent of Schisandra sphenanthera to protect against APAP-induced injury in LO-2 cells, it showed obviously anti-fibrotic effect in BDL and CCl4-induced liver fibrosis model mice, indicating the potential of SolB to be used in the treatment of liver fibrosis. In addition, Many studies have shown that SolB could inhibit liver injury by regulating multiple pathways (Jiang et al., 2015; Jiang et al., 2016; Lu et al., 2016; Zeng et al., 2017), these studies may help explain the target of SolB in the prevention and treatment of liver injury and the related mechanism.

Hepatic stellate cell activation and chronic inflammation are also important contributors to the development of liver fibrosis (Bataller and Brenner, 2005; Seki and Schwabe, 2015). TGF-β plays a critical role in the activation of HSCs, inhibiting TGF-β activity is a potential and effective treatment for hepatic fibrosis (Gyorfi et al., 2018; Dewidar et al., 2019). Our data showed that Wedelolactone (WeD), a coumarin isolated from Eclipta prostrate L exhibiting hepatoprotective effect and anti-fibrotic effect on human hepatic stellate cell line LX-2 (Xia et al., 2013; Lu et al., 2016; Luo et al., 2018), inhibited TGF-β-mediated induction of Smad3 and its phosphorylation, suggesting that WeD could directly target TGF-β/Smads signaling pathway to inhibit the activation of HSCs. Recent studies also demonstrate the role of WeD in anti-inflammation, WeD is an inhibitor of IKK that is critical for activation of NF-κB (Luo et al., 2018), it has also been reported to suppress NLRP3 infammasome activation (Wei et al., 2017; Cheng et al., 2019). Our data also confirmed the inhibitory effect of WeD on production of TNF-α and IL-1β in macrophages, which are involved in the progression of fibrogenesis through promoting survival and activation of HSCs (Gieling et al., 2009; Osawa et al., 2013; Pradere et al., 2013).

Hepatocyte injury, triggered by multifarious factors, is the main driver of chronic liver inflammation and fibrosis (Luedde et al., 2014; Seki and Schwabe, 2015). Meanwhile, previous studies have demonstrated that apoptotic hepatocyte could promote the secretion of pro-inflammatory and profibrogenic cytokines from macrophages, and to directly promote HSC activation (Canbay et al., 2003; Zhan et al., 2006; Seki and Schwabe, 2015). HSCs, which is the main executors of fibrogenesis, interact with hepatocytes, hepatic macrophages, lymphocytes and endothelial cells resulting in the promotion of fibrogenesis (Bataller and Brenner, 2005; Bansal and Chamroonkul, 2019). Moreover, it has been suggested that damage-associated molecular patterns (DAMPs), which are released by dead or damaged cells, may also directly or indirectly promote fibrosis (Luedde et al., 2014; Seki and Schwabe, 2015). Elevation in ALT and AST has been regarded as the indicators of hepatocyte injury (Luedde et al., 2014; Seki and Schwabe, 2015). In our study, we have detected the level of serum ALT and AST in CCl4/BDL-induced hepatic fibrosis mice, and the results suggested that SolB, WeD or the combination of SolB and WeD treatment reversed the increase in ALT and AST serum levels induced by CCl4 or BDL, suggesting that the treatment reduced hepatocyte injury *in vivo*.

Combination therapies that address two or more key molecular players and/or pathways are considered to hold much promise for treatment of liver fibrosis (Trautwein et al., 2015). Hepatocyte injury, inflammation, or hepatic stellate cells (HSCs) activation are important contributors to the development of liver fibrosis (Bataller and Brenner, 2005; Gieling et al., 2009; Seki and Schwabe, 2015)**.** In our studies, we show that SolB inhibited hepatocyte injury, while WeD blocked HSC activation and inflammatory cytokines production in macrophage. The inhibitory effect of the combination of SolB and WeD on hepatic fibrosis is superior to that of individual SolB or WeD treatments in CCl4-induced liver fibrosis model mice, as evidenced by the histopathological assessment, quantitative analysis of fibrogenesis and anti-fibrinogenic genes or the expression of proteins such as collagen I and *α*-SMA. Our study indicated that combination therapy with two compounds (SolB and WeD) targeting different pathways exhibit better anti-fibrotic effect. Taken together, our data demonstrate that SolB and WED reverses the progress of liver fibrosis by targeting different cells and pathways, the inhibitory effect of the combination of the two on hepatic fibrosis is superior to that of SolB or WeD alone. Combination of SolB and WeD may be a potential candidate for the prevention and treatment of liver fibrosis.

## Data Availability

The raw data supporting the conclusion of this article will be made available by the authors, without undue reservation.
