# Corrigendum: The Combination of Schisandrol B and Wedelolactone Synergistically Reverses Hepatic Fibrosis *via* Modulating Multiple Signaling Pathways in Mice

**DOI:** 10.3389/fphar.2021.777914

**Published:** 2021-10-07

**Authors:** Yongqiang Ai, Wei Shi, Xiaobin Zuo, Xiaoming Sun, Yuanyuan Chen, Zhilei Wang, Ruisheng Li, Xueai Song, Wenzhang Dai, Wenqing Mu, Kaixin Ding, Zhiyong Li, Qiang Li, Xiaohe Xiao, Xiaoyan Zhan, Zhaofang Bai

**Affiliations:** ^1^ Department of Hepatology, The Fifth Medical Centre, Chinese PLA General Hospital, Beijing, China; ^2^ Research Center for Clinical and Translational Medicine, The Fifth Medical Center of Chinese PLA General Hospital, Beijing, China; ^3^ China Military Institute of Chinese Materia, The Fifth Medical Centre, Chinese PLA General Hospital, Beijing, China

**Keywords:** schisandrol B, wedelolactone, hepatic fibrosis, combined pharmacotherapy, TGF-β1/Smads signaling pathway

In the original article, there was a mistake in Figure 5 as published. The symbols (*, **, ***, ns) of statistic differences in Figures 5B–F were wrongly labeled. The wrongly labeled Figure 5 was uploaded by accident when submitting the corrections of proof. The corrected Figure 5 appears below.

**FIGURE 5:**
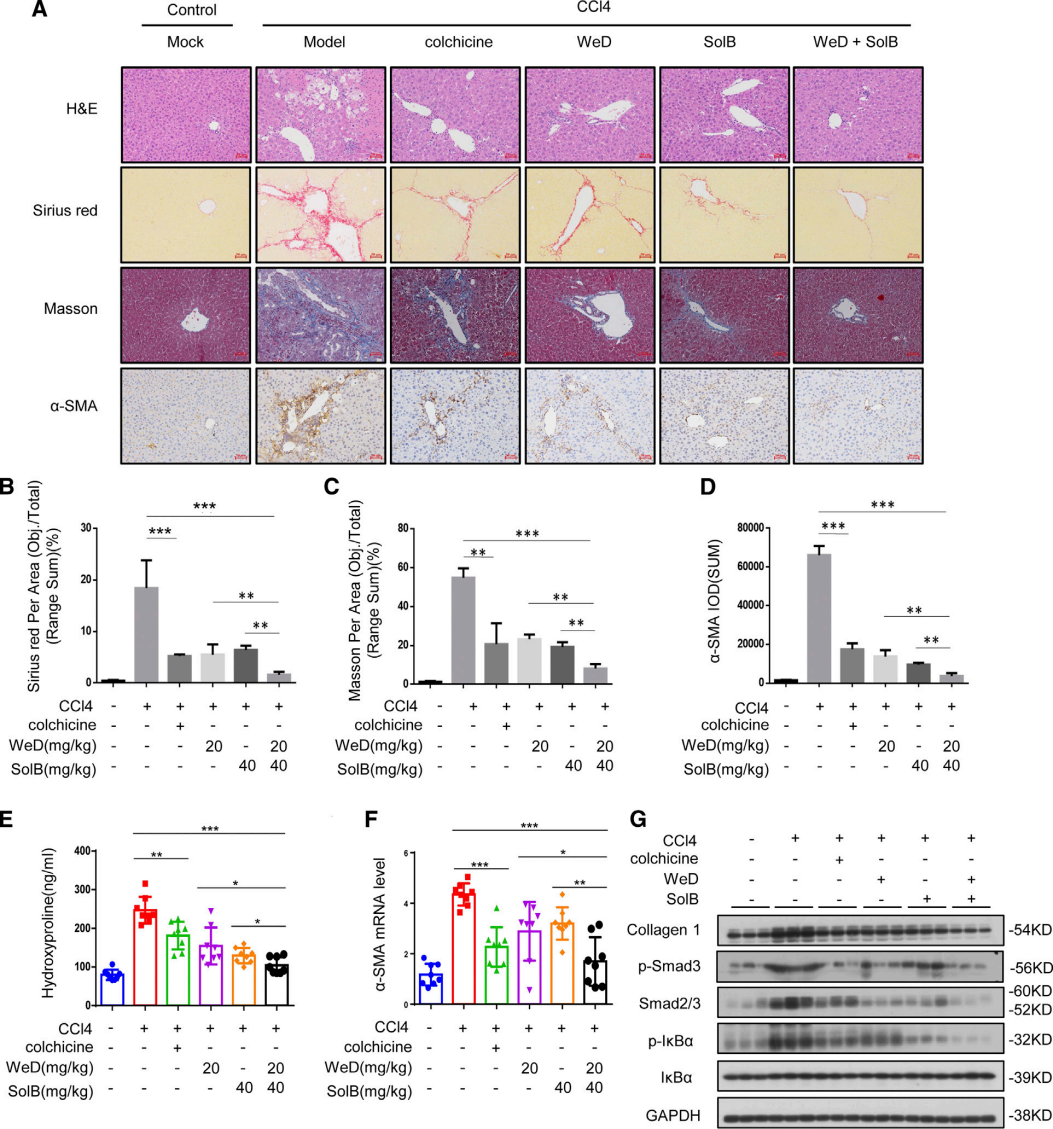
A combination of SolB and WeD treatment dramatically inhibits hepatic fibrosis and injury in CCL4-induced hepatic fibrosis mice. **(A)** Images of livers from control, CCL4-induced hepatic fibrosis mice, CCL4-induced hepatic fibrosis mice treated with colchicine(0.2 mg/kg), SolB(40 mg/kg), WeD(20 mg/kg) or combination of SolB(40 mg/kg) and WeD(20 mg/kg). Representative micrographs of liver H&E staining, Sirius red, Masson and α-SMA staining were shown. Scale bars represent 50 μm. **(B–D)** Quantitative results of Sirius red**(B)**, Masson**(C)** and α-SMA**(D)** staining sections. **(E)** Serum level of hydroxyproline of control, CCL4-induced hepatic fibrosis mice, CCL4-induced hepatic fibrosis mice treated with colchicine(0.2 mg/kg), SolB(40 mg/kg), WeD(20 mg/kg) or combination of SolB and WeD. **(F)** Quantitative PCR analysis of mRNA levels of α-SMA in livers from control, CCL4-induced hepatic fibrosis mice, CCL4-induced hepatic fibrosis mice treated with colchicine(0.2 mg/kg), SolB(40 mg/kg), WeD(20 mg/kg) or combination of SolB and WeD. **(G)** Western blot analysis of Collagen1, p-Smad3, Smad2/3, p-IκBα, IκBα and GAPDH in livers from control, CCL4-induced hepatic fibrosis mice, CCL4-induced hepatic fibrosis mice treated with colchicine(0.2 mg/kg), SolB(40 mg/kg), WeD(20 mg/kg) or combination of SolB and WeD. Data are expressed as Mean ± SD (n = 8 or 3 mice). Statistics differences were analyzed using One-way ANOVA followed by Tukey’s post hoc tes: *p < 0.05, **p < 0.01, ***p < 0.001. NS, no significance.

The authors apologize for this error and state that this does not change the scientific conclusions of the article in any way. The original article has been updated.

