# Supplementary material for: The Combination of Schisandrol B and Wedelolactone Synergistically Reverses Hepatic Fibrosis Via Modulating Multiple Signaling Pathways in Mice
Source: Front Pharmacol. 2021 Jun 3;12:655531. doi: 10.3389/fphar.2021.655531 (PMC8211319; doi:10.3389/fphar.2021.655531)
Supplement: Supplementary file 1 [file Presentation1.pdf]

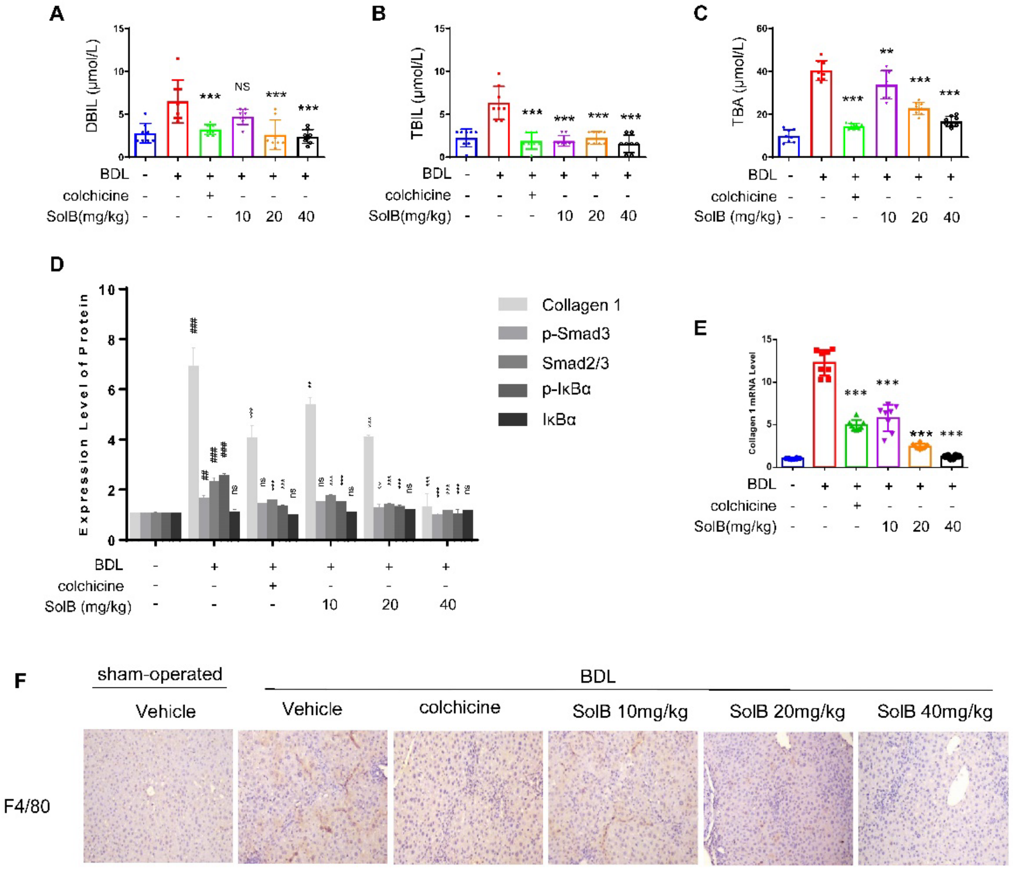

Figure S1. SolB attenuates hepatic injury and fibrosis induced by BDL in mice.

(A-C) Serum level of DBIL (A), TBIL (B) and TBA (C) of sham-operated mice (SHAM), bile duct-ligated mice (BDL) and BDL-mice treated with SolB or colchicine(0.2mg/kg). (n=8 mice) (D) Quantitative PCR analysis of mRNA levels of Collagen1 in livers from sham-operated mice, BDL mice, BDL-mice treated with SolB. (n=8 mice). (E) Quantitative results of western blot in livers from sham-operated mice, BDL mice, BDL-mice treated with SolB. (n=3 mice). (F) Representative micrographs of liver F4/80 staining were shown. Scale bars represent 100μm. Livers from sham-operated mice (SHAM), bile duct-ligated mice (BDL), BDL-mice treated with SolB. Data are expressed as Mean±SD. Statistics differences were analyzed using One-way ANOVA followed by Dunnett's test: #P<0.05, ##P<0.01, ###P<0.001 compared with the control group, *P<0.05, **P<0.01, ***P<0.001 compared with the model group (BDL). NS, not significant.

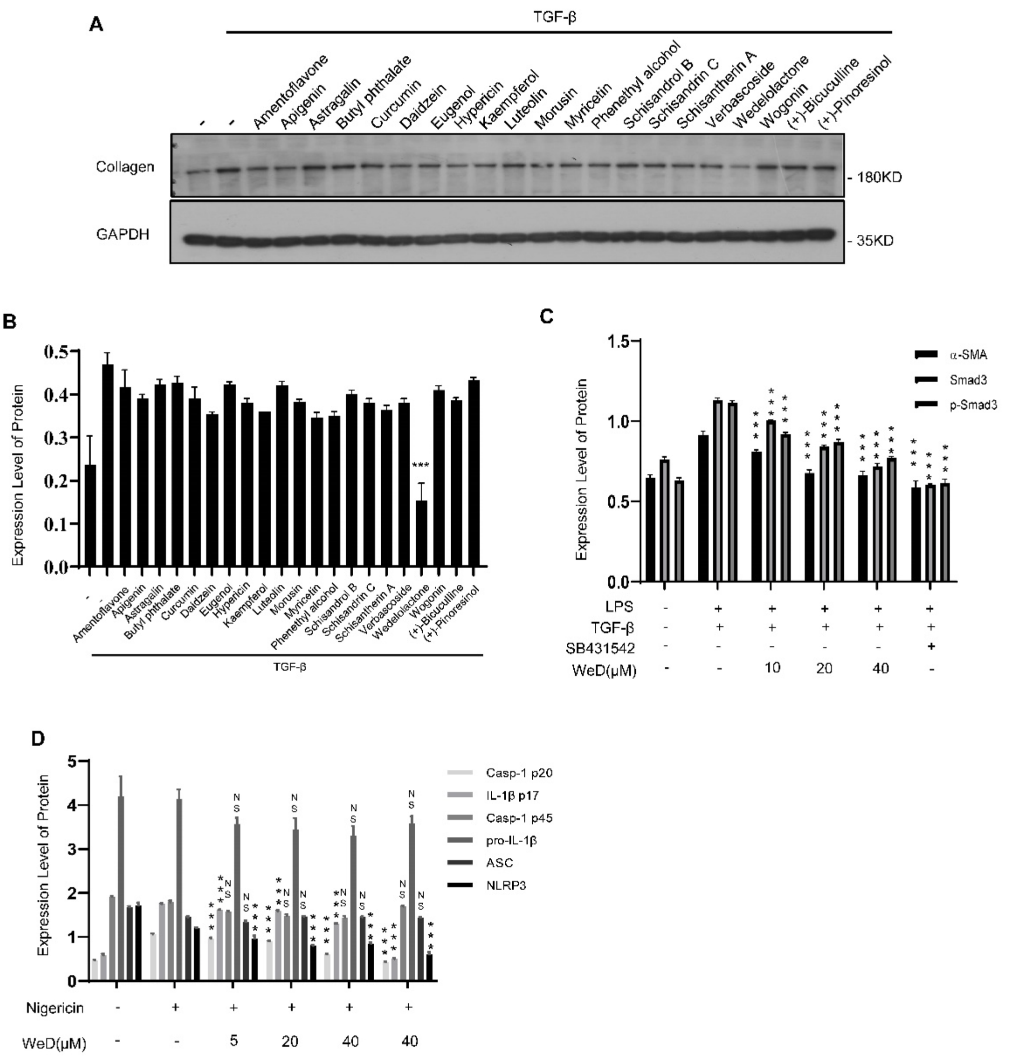

Figure S2. WeD inhibits TGF-β1/Smad-mediated activation of HSCs and blocks the production of TNF-α and IL-1β in macrophages. （A）Screening compounds that could inhibit TGF-β1/Smad signaling in LX-2 cells. (B) Quantitative results of western blot from α-SMA, Smad3, p-Smad3, GAPDH in LX-2 cells treated with WeD (10μM, 20μM, 40μM) and then stimulated with TGF-β1 (5ng/mL) combined with LPS (50ng/mL). (C) Quantitative results of western blot from caspase-1 (p20) and IL-1β in SN and pro- IL-1β, caspase-1 (p45), NLRP3 and ASC in WCL of LPS-primed BMDMs treated with WeD (5μM, 10μM, 20μM, 40μM) and then stimulated with nigericin. Statistics differences were analyzed using One-way ANOVA followed by Dunnett's test: #P<0.05, ##P<0.01, ###P<0.001 compared with the control group, *P<0.05, **P<0.01, ***P<0.001 compared with the model group (BDL). NS, not significant.

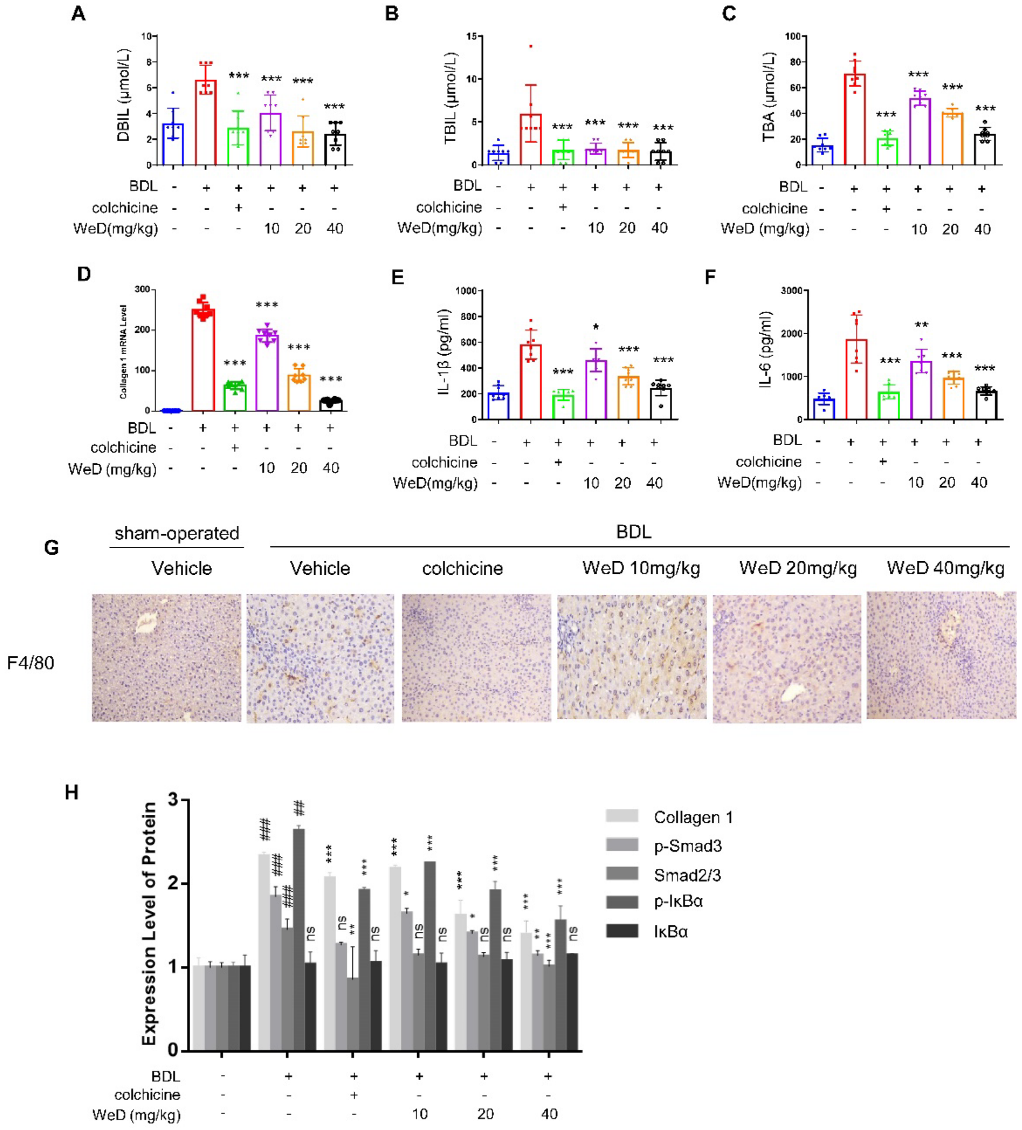

Figure S3. WeD attenuates hepatic injury and fibrosis induced by BDL in mice. Serum level of DBIL (A), TBIL (B) and TBA (C) of sham-operated mice (SHAM), bile duct-ligated mice (BDL) and BDL-mice treated with WeD or colchicine(0.2mg/kg). (n=8 mice). (D) Quantitative PCR analysis of mRNA levels of Collagen1 in livers from sham-operated mice, BDL mice, BDL-mice treated with WeD. (n=8 mice). (E and F) Serum level of IL-1β (E) and IL-6 (F) of sham-operated mice (SHAM), bile duct-ligated mice (BDL) and BDL-mice treated with WeD or colchicine(0.2mg/kg). (n=8 mice). (G) Representative micrographs of liver F4/80 staining were shown. Scale bars represent 100μm. Livers from sham-operated mice (SHAM), bile duct-ligated mice (BDL), BDL-mice treated with WeD. (H) Quantitative results of western blot in livers from sham-operated mice, BDL mice, BDL-mice treated with WeD (n=3 mice). Data are expressed as

Mean±SD. Statistics differences were analyzed using One-way ANOVA followed by Dunnett's test: *P<0.05, **P<0.01, ***P<0.001. NS, no significance.

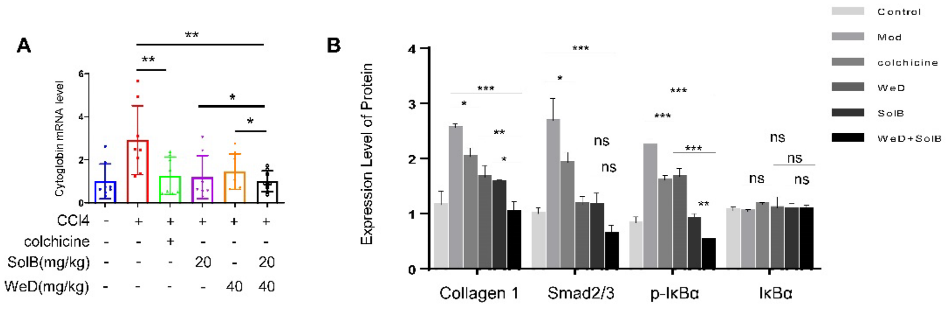

Figure S4. A combination of SolB and WeD treatment dramatically inhibits hepatic fibrosis and injury in CCL4-induced hepatic fibrosis mice. (A) Quantitative PCR analysis of mRNA levels of Cytoglobin in livers from control, CCL4-induced hepatic fibrosis mice, CCL4-induced hepatic fibrosis mice treated with colchicine(0.2mg/kg), SolB(40mg/kg), WeD(20mg/kg) or combination of SolB and WeD. Data are expressed as Mean±SD (n=8 mice). (B) Quantitative results of western blot analysis of Collagen1, Smad2/3, P-IKBα, IKBα and GAPDH in livers from control, CCL4-induced hepatic fibrosis mice, CCL4-induced hepatic fibrosis mice treated with colchicine(0.2mg/kg), SolB(40mg/kg), WeD(20mg/kg) or combination of SolB and WeD. Data are expressed as Mean±SD (n=3 mice). Statistics differences were analyzed using One-way ANOVA followed by Tukey's post hoc test: *P<0.05, **P<0.01, ***P<0.001. NS, no significance.

Full unedited gel for Figure 2H

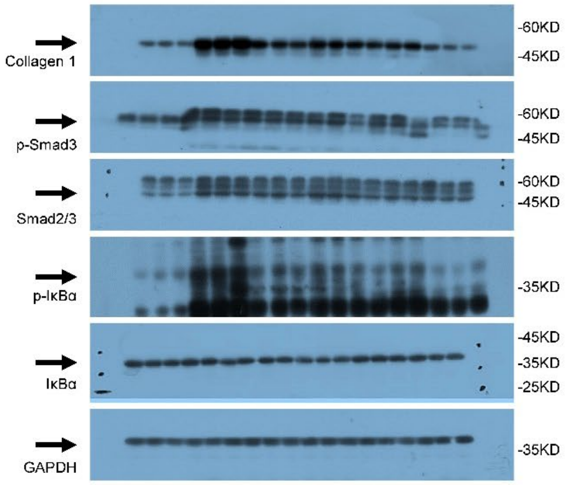

Full unedited gel for Figure 3A

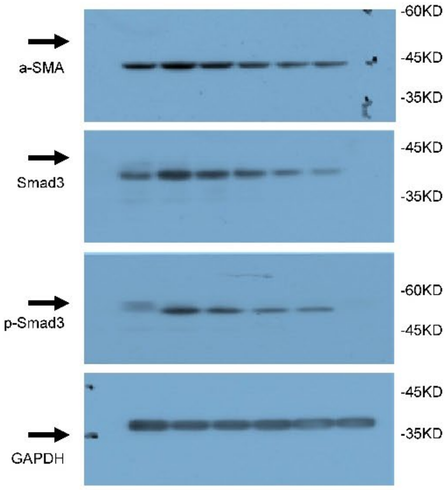

Full unedited gel for Figure 3E

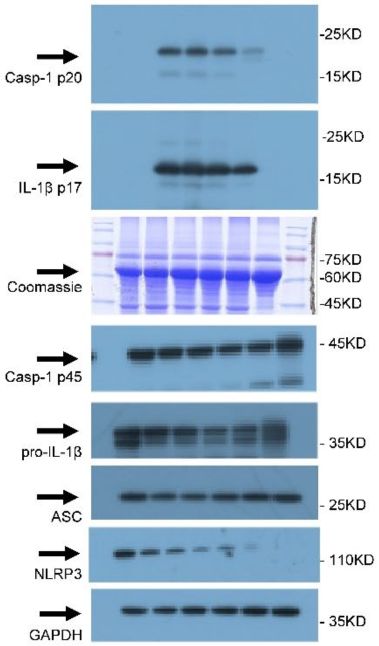

Full unedited gel for Figure 4H

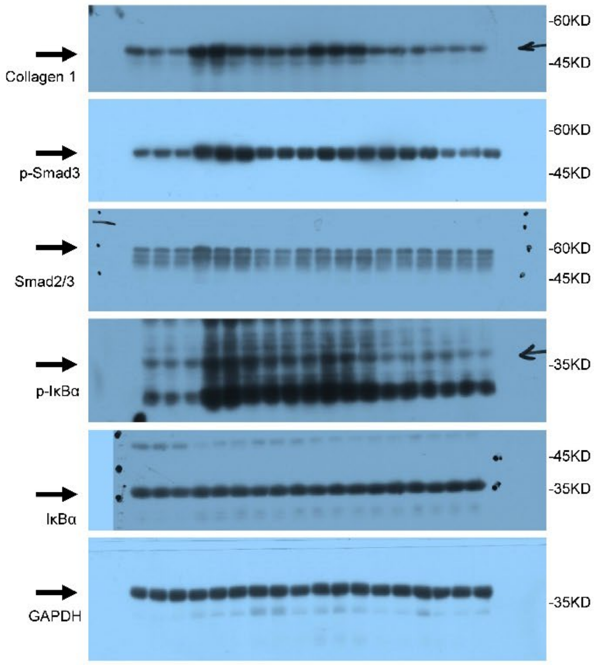

Full unedited gel for Figure 5G

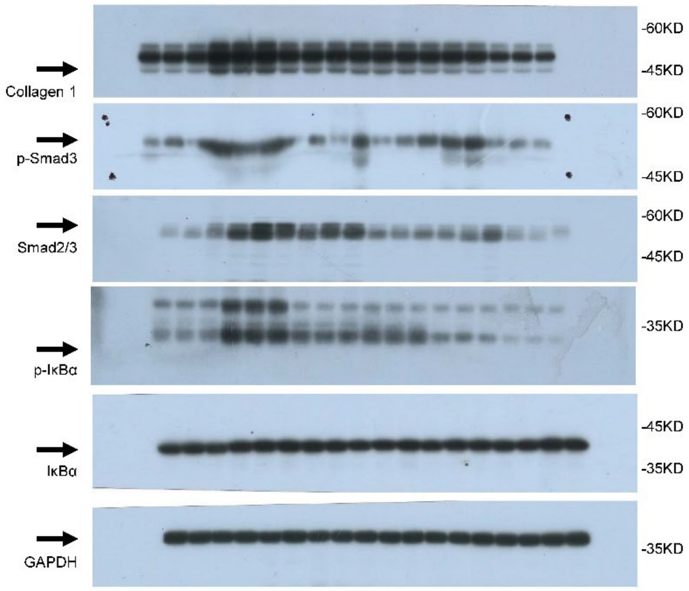

Full unedited gel for Figure S2A

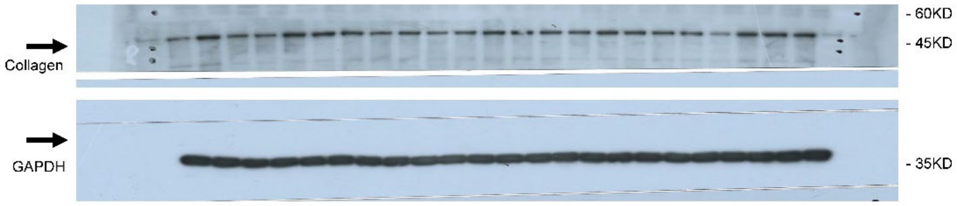

Figure S5. Full images of all the western blots.
